# How Croatian patients suffering from amyotrophic lateral sclerosis have been turned into medical tourists – a comment on a medical and social phenomenon

**DOI:** 10.3325/cmj.2014.55.443

**Published:** 2014-10

**Authors:** Dinko Mitrečić, Ervina Bilić, Srećko Gajović

**Affiliations:** 1 Laboratory for Stem Cells, Section of Neurogenetics, Cytogenetics, and Developmental Genetics, Croatian Institute for Brain Research, University of Zagreb School of Medicine, Zagreb, Croatia dominic@mef.hr; 2 Referral Center for Neuromuscular Diseases and Clinical Electromyoneurography, Department of Neurology, University Hospital Center Zagreb, University of Zagreb School of Medicine, Croatia; 3 Croatian Medical Journal, Zagreb, Croatia

In the last few weeks, Croatia has witnessed a rarely seen surge of public interest in neurologic disease amyotrophic lateral sclerosis (ALS). In a rather organized manner and with significant media support, money was raised to send Croatian patients suffering from ALS to China to receive a treatment referred to as “stem cell therapy.” Even before this action, individual Croatian patients, including children suffering from a variety of diseases, tried on their own to seek this type of “therapy.” What makes the current situation specific is that a subgroup of patients offered to another group of patients a financially covered “package” for a treatment in a hospital in China without consulting their doctors or other Croatian medical experts. Upon returning home, the patients, together with their doctors and families, are now facing consequences of this medical and social phenomenon.

This action seems to have appeared simultaneously with the viral social trend initiated in the USA, “the ALS ice bucket challenge,” ie, pouring ice-cold water over someone in combination with money donation for ALS associations (1). From a number of newspaper articles and TV broadcasts, one could get an impression that a competition in empathy was taking place. Many Croatian celebrities were challenged to spill a bucket of cold water over their heads in order to collect money for ALS patients. In parallel, medical laics representing several patient organizations appeared in prime time shows and talked about this “strange disease for which the only hope exists in stem cells clinics in Asia.” They expressed their intention to mobilize the nation to collect funds and send Croatian patients to China and registered non-profit organizations to raise funds. Using their own support groups and, to the best of our knowledge, without consultation with medical experts, they decided to send several patients from Croatia to one clinic in China, spending a minimum of EUR 50 000 per patient. This clinic appeared overnight as a place where ALS can be treated and even the national television broadcasted a show with a goal to collect the money to send the patients there.

ALS is the most common neurodegenerative disease of motor neurons, with a prevalence of 2-5/100 000 people. It is characterized by rapidly progressing dying of cortical, brainstem, and spinal motor neurons and remains an incurable disease, leading to a fatal respiratory failure on average within 5 years following the diagnosis. Although it is not our intention to take away somebody’s hope, it has to be clearly said that no one has ever been cured from this disease. Pathophysiology of ALS is complex and multifactorial. It includes mitochondrial dysfunction, intracellular protein aggregation, disturbances in RNA metabolism, impairment at the level of the axonal transport and at the neuromuscular synapse, blood-brain barrier breakdown, glial cell reaction/dysfunction and neuroinflammation. In 5%-10% of patients, ALS is inherited. In these cases, every fifth patient carries a detected mutation in superoxide dismutase 1 (SOD1), which has up to now been the most reliable and mostly used genetic model for ALS.

Although a national registry for ALS patients does not exist in Croatia, according to the Croatian Referral Center for Neuromuscular Disorders, the number of newly diagnosed ALS patients varies from 20 to 30 per year and the majority of these cases have sporadic ALS. Diagnosis of ALS is established according to the EFNS (European Federation of Neurological Societies) guidelines: it is based on anamnestic data, clinical presentation, electromyography, and on exclusion of other similar diseases. Differential diagnosis in a patient suspected for ALS is wide and the most common diagnoses established prior to the typical ALS development are cervical radiculomyelopathy and motor neuropathy (multifocal motor neuropathy or axonal motor neuropathy). Even though an absence of sensory alteration is mandatory for ALS diagnosis, in patients who previously had some kind of polyneuropathy (eg, diabetic sensory polyneuropathy) it may be difficult to recognize superimposed ALS. In some patients, only careful monitoring of clinical and electromyographic changes over a prolonged period may give the final diagnosis. The only drug prescribed to ALS patients is riluzole – in a standard dose of 50 mg twice a day.

Interestingly, ALS is probably the most common fear-causing disease among medical doctors and medical students. After a course in Neurology at the Medical School University of Zagreb, 12 medical students per academic year, which is 4% of the students in one generation, asked for a neurological exam and electromyography (unpublished data from the Registry of the Croatian Referral Center for Neuromuscular Disorders). We also observed that the number of patients with the diagnosis of benign fasciculation syndrome is dramatically rising, which is linked to the growing knowledge about ALS, including the recent “the ALS ice bucket phenomenon.”

One of the main premises and an essential prerequisite of good medical practice is that every new treatment, including new types of operation, new drugs, or new (stem) cell types, needs to be tested using well defined protocols. Every new treatment is thus first tested on experimental animals, where its potential for translation into clinical practice is evaluated. Data are collected by appropriate experimental procedure and subsequently published in peer reviewed journals. Clinical trials, as a continuation of preclinical results represent a long, step-wise procedure involving patients and healthy volunteers. If during clinical trials there is a money transfer involving patients, money can only be given to patients for their participation in early testing of procedure, and not taken away from them. Patients and their doctors need to be informed about all details of the procedure. In the case of cell therapy, the basic parameters include the source of stem cells, cell types (tested by set of various genetic and immunohistochemical markers), rate of cell differentiation prior to injection, cell number and viability, stem cell transplantation procedure, routes of administration, number of treatments, and a well-defined protocol for the treatment evaluation.

Based on successful preclinical trials, including published discoveries from our own group (first intravascular injection of stem cells into animals suffering from ALS) (2) – stem cell clinical trials on ALS patients have been launched. Currently, there are approximately 15 ongoing clinical trials in the EU and USA. Most of them are focused on intraspinal delivery of fetal/mesenchymal/neural stem cells (USA, Italy, Spain) and some use intrathecal delivery of autologous bone marrow-derived cells. One of the major principles of transparency is that every trial is registered and well described in a publically available database of clinical trials (3). Clinical trial registration is a prerequisite for publication of results.

Unfortunately, due to their high and very promising potential, stem cells are sometimes used in unwanted and non-ethical ways. This is not surprising since the cell therapy industry reached a year turnover of one billion dollars (4). Cell therapy-based medical tourism is a phenomenon when people suffering from fatal diseases search for help in countries that obviously allow or do not control “stem cell clinics.” There, patients can receive cell therapy for numerous diseases with only one prerequisite – if they pay for it.

The Croatian patients suffering from ALS who were received “stem cell treatment” in the clinic in China were treated in a way incompatible with ethical standards. Medical care for them practically did not exist. As many of them were in advanced stage of disease, they had to be accompanied by someone from Croatia to help them even with everyday care including basic hygiene. No one explained them anything about the ongoing procedures. The medical records that they brought with them to Croatia listed several treatments, the majority of which were very general suggestions (eg, to “stop smoking” or to “think positively”), but “stem cell therapy” was only mentioned by name, with no specifics given. For something that is so complex, specific, and potentially dangerous, this is completely unacceptable. The patients have to be informed about all the details of the procedure and they have the right that their medical doctors from their countries of origin can freely communicate with the doctors in those clinics. Omitting the detailed medical records could even be done on purpose since to patients secret therapies can sound attractive, promising, and almost magic. Unfortunately, they are only previously unevaluated procedures with unproved non-standardized products bearing a high risk of side effects.

To prevent such practices, International Society for Stem Cell Research published the Patient Handbook on Stem Cell Therapies (5). This brochure warns patients about a growing fraud in the field of stem cell therapy and advises that only FDA or EMEA registered procedures are reliable. The same message is brought by the Croatian Brain Council, the national coordination of 25 societies (6). The Croatian Brain Council gathers experts in all fields of brain research and brain therapy and represents an appropriate starting point for any action aimed toward less known or less standardized procedures. Stem cell medical tourism brings negative consequences for both patients and medical experts. While patients are in danger of complications arising from not well controlled procedures, hard work and continuous progress of researchers who adhere to the principles of good medical practice are in jeopardy to be declared slow and inefficient. The failure of unapproved and uncontrolled stem cell treatment can create a wrong impression that stem cell based therapy has no potential and that funding of stem cell research is not bringing results.

Although the authors understand the hope that all of a sudden a cure for ALS would appear, we express our concerns for patients. It is of vital significance that the medical community shares information about the disease, treatment possibilities, and new research trends to the public. Such evidence-based information needs to be used to confront the well marketed, immediate, costly, and potentially dangerous trend of “medical tourism” in search for stem cell therapies. The current example shows that the decisions about the patients are not only in the hands of the doctors, but that there are many stakeholders involved. This does not only include the patients and their families, but also the charities, media, politicians, and many other compassionate individuals. The complexity of the decision-making process turns the patients into “bioobjects,” a phenomenon explained in a recent series of articles in the *Croatian Medical Journal* (7,8).

Instead of investing wisely into further development of stem cell research and medical standards in Croatia, funds raised from Croatian citizens, already heavily hit by the ongoing financial crisis, were wasted due to the false promises and ignorance. Thus we faced two losses: the funds were spent in vain and the patients were put in jeopardy. Therefore, we urge that future action for ALS or other diseases should be coordinated in communication with the doctors who treat the patients and in consultation with experts who are working in this field. They have dedicated their lives to a major goal: to help and not to harm their patients.
